# In Vitro and In Vivo Antagonism of a G Protein-Coupled Receptor (S1P_3_) with a Novel Blocking Monoclonal Antibody

**DOI:** 10.1371/journal.pone.0035129

**Published:** 2012-04-05

**Authors:** Greg L. Harris, Michael B. Creason, Greg B. Brulte, Deron R. Herr

**Affiliations:** 1 Expression Drug Designs, LLC, San Marcos, California, United States of America; 2 Department of Biology, San Diego State University, San Diego, California, United States of America; Florida International University, United States of America

## Abstract

**Background:**

S1P_3_ is a lipid-activated G protein-couple receptor (GPCR) that has been implicated in the pathological processes of a number of diseases, including sepsis and cancer. Currently, there are no available high-affinity, subtype-selective drug compounds that can block activation of S1P_3_. We have developed a monoclonal antibody (7H9) that specifically recognizes S1P_3_ and acts as a functional antagonist.

**Methodology/Principal Findings:**

Specific binding of 7H9 was demonstrated by immunocytochemistry using cells that over-express individual members of the S1P receptor family. We show, in vitro, that 7H9 can inhibit the activation of S1P_3_-mediated cellular processes, including arrestin translocation, receptor internalization, adenylate cyclase inhibiton, and calcium mobilization. We also demonstrate that 7H9 blocks activation of S1P_3_ in vivo, 1) by preventing lethality due to systemic inflammation, and 2) by altering the progression of breast tumor xenografts.

**Conclusions/Significance:**

We have developed the first-reported monoclonal antibody that selectively recognizes a lipid-activated GPCR and blocks functional activity. In addition to serving as a lead drug compound for the treatment of sepsis and breast cancer, it also provides proof of concept for the generation of novel GPCR-specific therapeutic antibodies.

## Introduction

The use of monoclonal antibodies (mAbs) to antagonize transembrane receptors has met with tremendous clinical and commercial success over the course of the past decade. The success of antibody drugs is based on their exquisite specificity and affinity, which are essential components of targeted molecular therapy. With 23 antibody drugs currently approved for clinical use and annual sales in the tens of billions of dollars [1], these biologics are being used for a wide range of indications such as inflammatory diseases, autoimmune diseases, stroke, and heart disease, but the greatest therapeutic antibody success stories involve the treatment of cancer. Examples of some the most effective and widely used, anti-cancer therapeutic antibody drugs include trastuzumab (Herceptin®, a HER2 inhibitor), bevacizumab (Avastin®, a VEGF inhibitor), and panitumumab (Vectibix™, an EGFR inhibitor).

**Figure 1:**
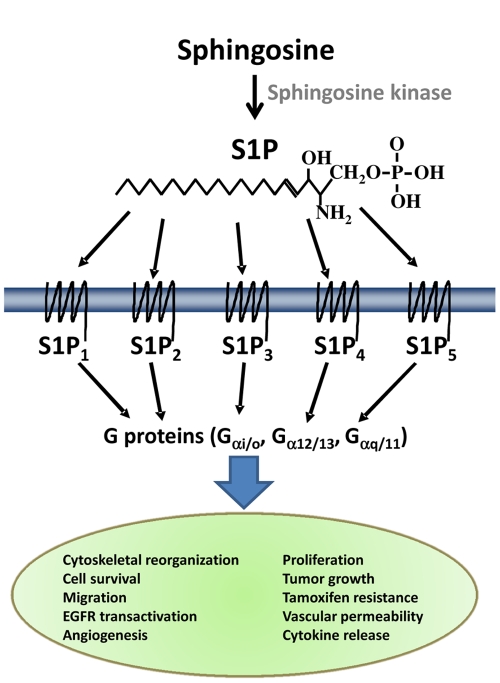
S1P signaling. S1P is an extracellular signaling molecule, generated by the phosphorylation of sphingosine, that exerts a variety of effects on a family of 5 cognate GPCRs.

Sphingosine 1-phosphate (S1P) is a lipid signaling molecule (Figure 1) that is present in serum at biologically relevant concentrations (high nanomolar range). S1P is generated by the phosphorylation of sphingosine by sphingosine kinase in the final step of a highly conserved metabolic pathway [2]. Although there have been reports of some intracellular roles of S1P [3]–[5], the majority of its effects are mediated by a family of five known S1P-selective G protein-coupled receptors (GPCRs). These receptors belong to a GPCR subfamily (formerly known as the “Edg” receptors) whose members are activated by S1P (S1P_1–5_) or the structurally similar lipid, lysophosphatidic acid (LPA; LPA_1–3_). They couple to a number of G proteins and downstream effectors to elicit a variety of responses in almost every known cell type. The responses vary among cell types depending on the expression profile of the receptors and effectors, but notably include proliferation, survival, and cytoskeletal rearrangement (reviewed in: [6]–[9]).

Previous studies are consistent with a pro-tumorigenic role of S1P. S1P is known to increase the proliferation, survival, motility, and invasiveness of breast tumor cells [10]–[13]. In addition, the known involvement of S1P signaling in the processes of angiogenesis and vascular maturation underscores the importance of this pathway in cancer progression [14], [15]. The tumor-promoting effect of S1P is directly supported by the observation that overexpression of sphingosine kinase in MCF-7 cells promotes tumorigenesis and tumor vascularization in a nude mouse model [10]. Furthermore, it has been demonstrated that neutralization of S1P has a potent tumor-suppressive effect [16], an approach that is currently under clinical investigation.

In breast cancer cells (BCCs) the tumorigenic effects of S1P are likely to be largely mediated by the activation of cognate receptor subtype S1P_3_. S1P_3_ is the most highly expressed S1P receptor in BCCs [17], [18], is known to promote cell migration [19]–[21] and EGF responsivity [11], [12], [18], [22], [23], and may mediate the proliferative effects of estrogen [24]. In addition to the direct effects that S1P_3_ have on BCCs, S1P_3_ also mediates angiogenesis [25]–[31], thus promoting tumor growth by increasing vascularity. A recent study provided evidence for the clinical importance of S1P_3_ by showing that expression of S1P_3_ in breast tumors positively correlates with decreased tamoxifen sensitivity and decreased patient survival [32].

In addition to promoting tumor development, activation of S1P_3_ is also involved in the pathology of inflammatory responses. This is most clearly illustrated by the fact that mice specifically lacking S1P_3_ are resistant to the effects of the bacterial endotoxin lipopolysaccharide (LPS) [33]. There is near complete attenuation of inflammatory cytokine release in S1P_3_^-/-^ mice following LPS challenge. Most importantly, when LPS is administered at a dose that is lethal to 90% of wild-type mice, more than 80% of S1P_3_ knockouts survive. The protective effect of S1P_3_ loss-of-function is likely due to the roles of S1P_3_ in both immune cells and endothelial cells. S1P_3_ has been shown to mediate pro-inflammatory responses in a number of pathological conditions [34]. This is mediated by multiple immune cell types including dendritic cells [35], [36]. Moreover, it is known that activation of S1P_3_ on endothelial cells causes the disruption of tight junctions and an increase in vascular permeability that results in hemodynamic instability during septic shock [37]–[40]. This pathological activation of S1P_3_ occurs downstream of thrombin-induced innate immune responses to cause an amplification of cytokine release and inflammation and a loss of vascular integrity [33], [41].

Considering these known pathological roles, we reasoned that specific antagonism of S1P_3_ would be likely to provide effective protection against breast tumor development and acute systemic inflammation. Since the structural similarities among the “Edg” family of receptors provide significant challenges to the generation of subtype-selective small molecule inhibitors, we elected to pursue the development of a blocking monoclonal antibody. While this approach itself has unique challenges for GPCR targets, we have developed one S1P_3_-blocking mAb: 7H9. Here we demonstrate the specificity and efficacy of 7H9 and evaluate its therapeutic potential in animal models for sepsis and breast cancer.

## Results

### 7H9 Specifically Binds S1P_3_

**Figure 2:**
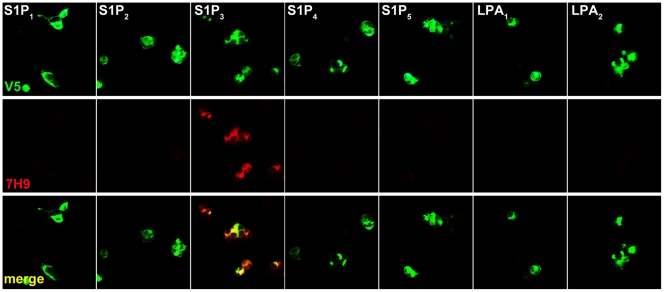
mAb 7H9 specifically recognizes S1P_3_. HEK293 cells were transfected with expression constructs for all closely related “Edg” family GPCRs (green), fixed, incubated with purified 7H9 (1 µg/ml), and visualized with a Cy3-conjugated secondary antibody (red). 7H9 specifically recognizes cells expressing S1P_3_, but not its closest homologs.

Monoclonal antibody 7H9 was evaluated for specificity to S1P_3_ by immunocytochemistry (Figure 2). S1P_3_ is a member of the highly conserved “Edg” family of GPCRs. HEK293 cells were transfected with expression constructs for each of 7 different “Edg” family receptors, then fixed and incubated with purified 7H9 antibody (1 µg/ml). Specific binding to S1P_3_-expressing cells was apparent by epifluorescence, while binding to the remaining “Edg” receptors was undetectable.

### 7H9 Blocks Activation of S1P_3_

Since stimulation of S1P_3_ results in the activation of multiple downstream processes, we evaluated 7H9 for its ability to inhibit a number of distinct S1P_3_-mediated events.

#### Arrestin translocation

**Figure 3:**
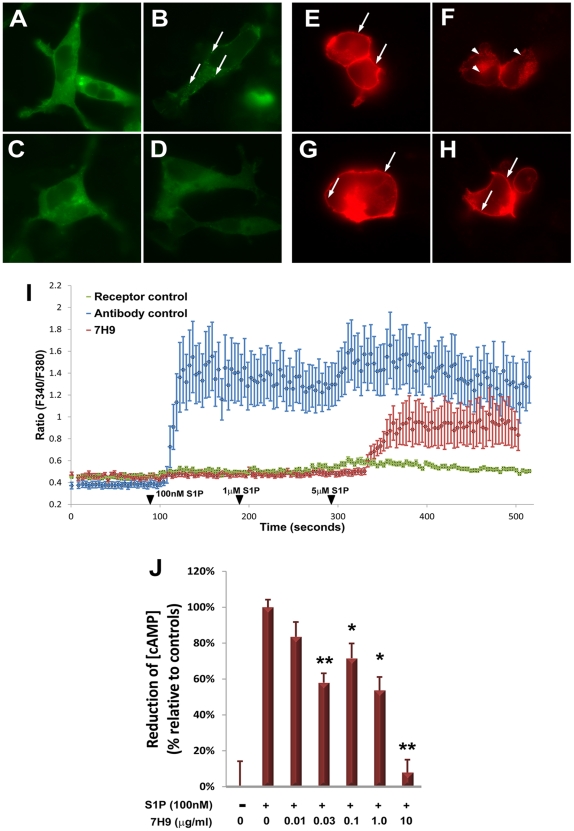
7H9 blocks activation of S1P_3_. (A-D) 7H9 blocks S1P_3_-mediated arrestin translocation. β-arrestin (green) is cytosolic in quiescent cells and appears as diffuse labeling (A). Following stimulation with 1 µM S1P (B), arrestin translocates to the plasma membrane and is rapidly internalized into intracellular vesicles (arrows). In contrast, when cells are pre-treated with 1 µg/ml 7H9 arrestin localization is diffuse and cytoplasmic in both the absence (C) and presence (D) of 1 µM S1P. (E-H) 7H9 blocks S1P-dependent internalization of S1P_3_. Epitope-tagged S1P_3_ is normally abundant on the plasma membrane (E, arrows), but is internalized into intracellular vesicles (arrowheads) upon stimulation with 1 µM S1P (F). Following pre-treatment of cells with 7H9, S1P_3_ remains localized to the plasma membrane in the absence (G) or presence (H) of 1 µM S1P. (I) 7H9 blocks S1P_3_-dependent calcium mobilization. S1P_3_-expressing cells exhibited increased intracellular [Ca^2+^] upon stimulation with 100 nM S1P (blue, antibody control). Cells within the same culture that did not express S1P_3_-EGFP (green, receptor control) showed no change in [Ca_i_^2+^]. Similarly, cells expressing S1P_3_ that were pre-treated with 7H9 (red, 7H9) also showed no response to 100 nM and 1 µM S1P, but gave a partial response at 5 µM. (J) 7H9 blocks S1P_3_-dependent inhibition of AC. cAMP was measured in S1P_3_-expressing cells by ELISA and normalized to controls. The graph represents the change in cAMP content from unstimulated cells, relative to the change observed in stimulated cells with no 7H9 pre-treatment. *p<0.05, **p<0.01. Error bars  =  S.E.M.

The signal amplitude of canonical GPCRs is limited by the arrestin family of regulatory proteins. Upon activation of GPCRs, cytosolic arrestin rapidly binds the receptor, stimulating receptor phosphorylation and subsequent clathrin-dependent internalization of the protein complex into intracellular endosomal vesicles. Vesicular localization of arrestin is indicative of GPCR activation. This can be visualized with the use of fluorescently labeled arrestin constructs [42]. To determine if 7H9 could inhibit S1P_3_-mediated arrestin translocation, we co-transfected HEK293 cells with epitope-tagged S1P_3_ and β-arrestin-EGFP (Figure 3A-D). EGFP labeling was diffuse throughout the cytoplasm of unstimulated cells (Figure 3A), but accumulated into distinct puncta 5 minutes after stimulation with 1µM S1P (Figure 3B). This subcellular redistribution was almost completely blocked by pre-incubation of cells with 7H9 (Figure 3C-D).

#### Receptor internalization

S1P_3_, like most canonical GPCRs, is rapidly internalized into cytoplasmic vesicles upon activation [43]. We investigated whether ligand-dependent internalization of S1P_3_ could be blocked by 7H9 (Figure 3E-H). RH7777 cells were transfected with epitope-tagged S1P_3_ constructs, stimulated with 1µM S1P for 15 minutes, fixed, and evaluated by immunocytochemistry. In unstimulated cells, S1P_3_ is largely localized to the plasma membrane (Figure 3E). After exposure to S1P, labeling becomes punctate and accumulates in the cytoplasm (Figure 3F). This process is dramatically reduced by pre-treatment with 7H9 (Figure 3G-H).

#### Calcium mobilization

S1P_3_ is a G_αq_-coupled GPCR and, upon stimulation with S1P, causes an increase in cytoplasmic Ca^2+^ concentration due to a release of intracellular Ca^2+^ stores [44], [45]. We investigated whether S1P_3_-dependent calcium release could be blocked by 7H9 (Figure 3I). RH7777 cells were transfected with S1P_3_-EGFP fusion constructs, stimulated with S1P, and evaluated for [Ca_i_^2+^] with calcium indicator dye fura-2. S1P_3_-expressing cells demonstrated a robust increase in [Ca_i_^2+^] upon exposure to 100 nM S1P, while S1P_3_-negative cells in the same culture showed no response. When cells were pre-incubated with conditioned media from 7H9 hybridoma cultures, S1P was unable to elicit the release of Ca_i_^2+^ until very high, non-physiological concentrations were applied.

#### Adenylate cyclase (AC) inhibition

S1P_3_ also couples to G_αi_, and consequently causes the inhibition of AC [45], [46]. This results in a reduction of intracellular cyclic adenosine monophosphate (cAMP). We investigated whether 7H9 could block S1P_3_-mediated G_αi_ signaling by measuring S1P-dependent changes in intracellular cAMP (Figure 3J). Stimulation of S1P_3_-expressing cells with 100 nM S1P resulted in a 35% decrease in intracellular [cAMP]. This effect was partially inhibited by pre-treatment with low concentrations of 7H9, and completely blocked at 10 µg/ml.

### 7H9 Ameliorates Systemic Inflammation In Vivo

**Figure 4:**
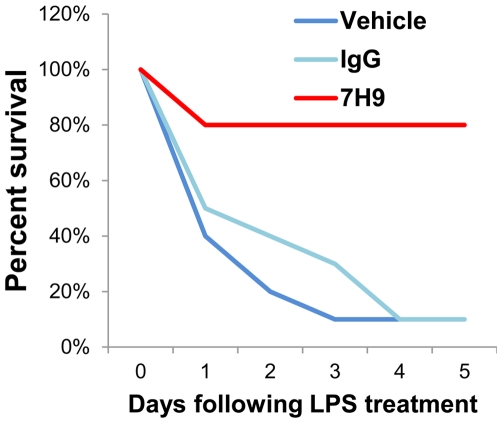
7H9 prevents lethality caused by LPS administration. Survival curve of mice treated with LPS (8 mg/kg, i.p.) on day 0. Mice were pre-treated with vehicle (PBS), IgG (normal mouse IgG), or 7H9.

Recent studies in mice demonstrated that deletion of the gene encoding S1P_3_ conferred resistance to the systemic inflammatory response induced by LPS, and afforded marked protection against the resulting lethality characteristic of disseminated intravascular coagulation (DIC) [33], [41]. We expected that similar protection would be obtained by antagonizing S1P_3_ in vivo with the administration of 7H9 (Figure 4). Mice were pre-treated with vehicle, non-specific mouse IgG, or 7H9; then were treated with LPS at the reported LD_90_ dose [33]. Consistent with previous studies, 9 out of 10 mice (90%) died within 4 days of LPS administration in both control groups. However, mice pre-treated with 7H9 were significantly protected, with only 2 of 10 mice (20%) succumbing to systemic inflammation.

### 7H9 Inhibits the Development of Breast Tumor Xenografts

**Figure 5:**
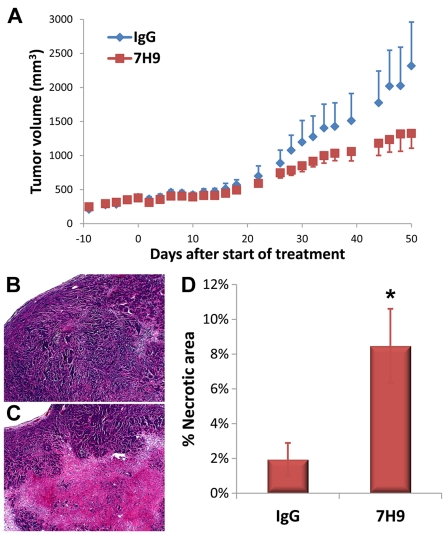
7H9 inhibits development of breast tumor xenografts. (A) Volumes of subcutaneous MCF7 tumors in nude mice were determined by caliper measurement. (B-C) Photomicrographs showing H&E stained tumors from IgG control (B) and 7H9-treated (C) mice. (D) Volumes of “necrotic” regions were determined by measuring their cross-sectional areas relative to total area of section. *p<0.05.

Since activation of S1P_3_ is known to contribute to the development and aggressiveness of breast tumors, we investigated whether antagonism of S1P_3_ with 7H9 could inhibit the progression of human breast tumor xenografts in mice (Figure 5). Solid tumors were established by subcutaneous implantation of MCF7 cells in Foxn1^Nu/Nu^ mice using standard techniques [47]–[49]. When tumors reached ∼200 mm^3^ by caliper measurement, mice were randomized into 2 groups and treated with normal mouse IgG or 7H9 (1 mg/kg, i.p., QOD), resulting in the stable accumulation of 7H9 in the serum at concentrations that were effective in vitro (Figure S1). After approximately 3 weeks of treatment, tumors in the 7H9 treatment group, on average, demonstrated a detectable decrease in the rate of development relative to the normal IgG control group (Figure 5A). This difference continued to broaden over the next few weeks but fell short of statistical significance (p = 0.16) when the experiment was terminated on day 50. Interestingly, however, upon histological examination we noticed a visible increase in the “necrotic” regions of the 7H9-treated tumors (Figure 5B-C). Quantification revealed that this was a statistically significant, 4-fold increase (Figure 5D).

## Discussion

While the use of therapeutic mAbs has achieved remarkable clinical success, the application of this technology to GPCR drug targets has been lagging, due primarily to the difficulty in generating selective antibodies for critical motifs in these complex, membrane spanning proteins. This is particularly problematic for the many GPCRs with *lipid* ligands, which typically have inaccessible transmembrane binding pockets. Here, we describe the generation of the first-reported mAb that selectively binds a lipid-activated GPCR, and blocks functional activity.

The strategy underlying the design of 7H9 involved the generation of an antibody that would bind an extracellular loop that formed the opening of the ligand binding pocket, thus preventing ligand-receptor interaction by steric hindrance. This is consistent with the results of the calcium assay (Figure 3I) in which normal, physiological concentrations of S1P had no effect, but unnaturally high concentrations of S1P (∼10-fold higher than normal serum levels) could overcome the inhibition. While this likely plays a role in the blocking effect of 7H9, the results of the functional assays suggest that the mechanism of action is more complex. Specifically, 7H9 at a concentration of 1 µg/ml (∼7 nM) provided efficient antagonism in arrestin, internalization, and calcium assays (Figure 3A-I), but a higher concentration was required for complete inhibition of the cAMP response (Figure 3J). While this may simply be the result of variation between experimental systems, it is possible that 7H9 acts as a somewhat *biased* antagonist, for example, by altering the confirmation of S1P_3_ in a way that decreases its affinity for G_αq_ to a greater extent than that of G_αi_. Additional functional studies with greater quantitative resolution have been planned to distinguish between these possibilities.

While the functional studies provide an initial demonstration of S1P_3_ antagonism, the best evidence for efficacy was provided by the in vivo studies. Notably, administration of 7H9 faithfully phenocopied the genetic null *S1pr3^-/-^* mouse in the LPS challenge, using the discrete and unambiguous readout of animal survival (Figure 4). While the effect of 7H9 in the xenografts was less discrete, there was a trend toward inhibition of tumor growth with 7H9 administration (Figure 5A). The fact that this did not reach statistical significance may be due to a number of factors. We expect the direct effect of S1P_3_ antagonism to be growth-inhibitory rather than cytotoxic, therefore, the timeframe of this model may be too short before significance was reached. After 50 days, the tumors reached a mass that necessitated euthanasia. Alternatively, the extent of the tumor-suppressive effect of 7H9 may not have been completely represented by measurement of tumor volume alone. Indeed, this is supported by the histological analysis of the tumors (Figure 5B-D). As was previously reported, disruption of S1P signaling causes an increase in necrotic lesions in tumor xenografts [16], likely due to decreased tumor angiogenesis.

The recent FDA approval of an S1P receptor-modulating drug for the treatment of multiple sclerosis [50] has brought a great deal of attention to this sub-class of GPCRs in the context of drug development. Significant evidence supports the idea that specific antagonism of S1P_3_ should be highly effective in treating a number of diseases including sepsis [33], [41], and breast cancer [32]. The interaction between S1P_3_ and estrogen signaling suggests that such a treatment would be particularly effective as co-therapy with tamoxifen for the treatment of breast cancer [32], [51]. This is consistent with our preliminary observations and is the subject of ongoing study. Our initial pilot study shows that 7H9 may enhance the effect of tamoxifen treatment (Figure S2). While there was no additive effect of combined 7H9/tamoxifen treatment on tumor *volume* (Figure S2A), the addition of 7H9 resulted in a further decrease in the number of mitotic cells in the tumor relative to tamoxifen alone (Figure S2B). Furthermore, there is also a trend toward increased tumor necrosis with combination therapy (Figure S2C). The fact that this parameter did not reach statistical significance is likely due to the small cohort size (N = 3) and awaits validation.

Since 7H9 is the only known high-affinity, S1P_3_-selective antagonist, this mAb is a viable candidate for development as a lead drug compound. It is also unlikely to be associated with significant adverse side effects. This is based on 3 lines of evidence: 1) Mice treated with 7H9 in this study exhibited no gross signs of toxicity, 2) S1P_3_ knockout mice are phenotypically indistinguishable from wild-type littermates [46], and 3) FTY720, a functional antagonist for multiple S1P receptors including S1P_3_, is well-tolerated in humans [52], [53]. Interestingly, administration of FTY720 has been shown to inhibit tumor growth and angiogenesis in vivo [54], [55], however, this compound acts as a potent immunomodulator through its action on S1P_1_. While this effect contributes to its efficacy in the treatment of multiple sclerosis, it is highly undesirable in cancer patients for which intact immune surveillance acts to prevent the development of metastatic lesions.

The identification of 7H9 demonstrates that we have developed a technology that is capable of generating specific blocking antibodies for an important but problematic class of GPCR drug targets. We are currently pursuing the development of mAbs for additional lipid-activated GPCRs to show that this approach may be generalized to multiple high-value targets.

## Materials and Methods

### Ethics Statement

Animal protocols were approved by the Institutional Animal Care and Use Committee at San Diego State University (protocol #: 10-01-00H and 11-02-003H) and conform to National Institutes of Health guidelines and public law. All efforts were made to minimize suffering.

### Reagents

Sphingosine-1-phosphate, D-erythro (S1P, BML-SL140-0001, Enzo Life Sciences, Inc, Farmingdale, NY, USA) was maintained as a 1mM stock solution in methanol and stabilized by diluting 1∶10 with 10% fatty acid-free bovine serum albumin (BSA) before making final working concentrations by diluting with Dulbecco’s modified Eagle medium (DMEM).

### Generation of 7H9

S1P_3_-blocking antibody 7H9 was generated by immunizing mice with a synthetic peptide (KKTFSLSPTVWFLREG) and generating hybridoma clones using standard protocols. Clones were selected with a proprietary screening platform. Out of 2,592 candidates, one clone (7H9) demonstrated suitable S1P_3_-specific binding. Unless otherwise indicated, all studies were performed using antibody that was purified from ascites fluid by protein A/G affinity chromatography (Thermo Fisher Scientific, Rockford, IL, USA).

### Cell Lines and Tissue Culture

HEK293 (CRL-1573), RH7777 (CRL-1601), C6 (CCL-107), and MCF7 (HTB-22) cells were obtained from American Type Culture Collection (ATCC). All cell lines were grown as monolayers in humidified incubators at 37°C with 5% CO_2_. Cells were cultured in DMEM, 25mM D-glucose, 4mM L-glutamine, 1 u/ml penicillin, 1 µg/ml streptomycin, 10% fetal bovine serum (Invitrogen, Carlsbad, CA, USA).

### Immunocytochemistry

For all studies involving immunolabeling, cells were cultured on collagen-coated coverslips (cat #08-115, Millipore, Billerica, MA, USA), fixed with 4% paraformaldehyde, incubated overnight with mouse monoclonal anti-V5 (cat #R960-25, Invitrogen, Carlsbad, CA, USA) diluted 1∶500 in blocking solution (phosphate buffered saline (PBS)/2.5% BSA/0.3% Triton X-100), then incubated with Cy3-anti-mouse (cat #AP192C, Millipore, Billerica, MA, USA) diluted to 1 µg/ml in blocking solution.

### Arrestin Translocation

Enhanced green fluorescent protein (EGFP) was cloned in frame to the C-terminus of full length human β-arrestin in vector pcDNA3.1 to generate a β-arrestin-EGFP fusion protein expression construct. Epitope-tagged S1P_3_ was generated by amplification of S1P_3_ from cDNA derived from MCF7 breast cancer cells (cat #HTB-22, ATCC) by polymerase chain reaction, and cloning into pcDNA3.1?V5-His TOPO (cat #K4800-01, Invitrogen, Carlsbad, CA, USA) per protocol. These constructs were co-transfected into HEK293 cells with Lipofectamine 2000 (Invitrogen, Carlsbad, CA, USA) per protocol. Cells were incubated in serum-free media 4 to 16 hours prior to stimulation with S1P. Cells were treated with 1mM S1P or BSA for 15 minutes, fixed and processed for immunocytochemistry as described above, and visualized by fluorescence microscopy. Expression of S1P_3_ was verified in all cells before capturing images of GFP localization.

### Ca^2+^ Imaging

Changes in intracellular [Ca^2+^] were determined using the ratiometric calcium indicator dye Fura-2. RH7777 cells were transfected with an S1P_3_-EGFP fusion expression construct, cultured on collagen-coated coverslips, loaded with Fura-2 acetoxymethyl ester (Fura-2/AM; 8µM) in the presence of 1.5 µM pluronic acid F-127, and incubated for 30–45 min at room temperature in the dark in DMEM. Coverslips were then washed with PBS and secured to the bottom of a laminar flow perfusion chamber RC-25 (Warner Instrument Corporation) with vacuum grease. Cells were selected for absence or presence of S1P_3_ expression based on EGFP fluorescence. S1P was prepared as described above and applied with a micropipette. Images of Fura-2-loaded cells with the excitation wavelength alternating between 340 nm and 380 nm were captured with a cooled CCD camera every ∼4 sec. The ratio of fluorescence intensity emitted after excitation at 340 nm and 380 nm (F340/F380) was calculated (MetaFluor, Molecular Devices). Cells that exhibited oscillatory fluorescence or elevated F340/F380 ratios (>0.7) prior to S1P stimulation were eliminated from analysis. N = 23–34.

### Camp Measurements

S1P_3_-EGFP over-expressing cell lines were generated by transfecting C6 glioma cells with linearized S1P_3_-EGFP-pcDNA3.1 using Lipofectamine 200 transfection reagent (Invitrogen, Carlsbad, CA, USA). Stable transfectants were selected using 1 mg/ml of geneticin (Invitrogen, Carlsbad, CA, USA) and clonally expanded. Cells were seeded at 50,000 cells/well in a 24-well plate, serum-starved overnight, pre-treated with 7H9 and S1P as indicated, and stimulated with 0.5 µM forskolin, 0.5 µM 3-isobutyl-1-methylxanthine to elevate basal cAMP. After a 20 minute incubation, cAMP content was determined from cell lysates by ELISA (Cayman Biochemicals, Ann Arbor, MI) per manufacturer’s instructions.

### Systemic Inflammation Model

Systemic inflammation experiments were performed on 6 week-old, male C57Bl/6J mice obtained directly from The Jackson Laboratory (Bar Harbor, Maine, USA). Working solutions of normal mouse IgG (cat # 31202, Thermo Fisher Scientific, Rockford, IL, USA) and 7H9 were made in PBS at 0.1 mg/ml, and were administered at 10 µl/g body weight, i.p., 3 times with the following schedule: 12 hours prior, 15 minutes prior, and 24 hours following LPS administration. LPS was prepared in a working solution of 0.8 mg/ml in PBS and administered once at 10 µl/g body weight i.p. Mice were monitored continuously for 1 hour, then 3 times/day for 5 days.

### Breast Tumor Xenografts

MCF7 human breast tumor cells were cultured until confluence and the formation of domes, then detached with a rubber policeman and suspended in 50%PBS/50% Matrigel (cat #356234, BD Biosciences, San Jose, CA, USA) at ∼25 × 10^6^ cells/ml. 100µl of cell suspension was delivered subcutaneously into 6 week-old, female NU/J mice (stock # 002019, The Jackson Laboratory, Bar Harbor, Maine, USA) under isoflorane anesthesia. Concurrently, a 60-day release estrogen pellet (cat #SE-121, Innovative Research of America, Sarasota, FL, USA) was implanted at a second subcutaneous site. Tumor development was monitored every ∼2 days by caliper measurement. Tumor volume was calculated by length × width^2^.

### Histology

Tumors were harvested, cut into pieces <10 mm in diameter, fixed in 4% paraformaldehyde overnight, processed and embedded in paraffin using standard techniques, sectioned at 10 µm, and stained with hematoxylin and eosin. Images were captured with a 5X objective. Area of necrotic lesions and total tumor area were determined with Image J software.

## Supporting Information

Figure S1 **Immunocytochemical analysis of serum from treated mice demonstrates that 7H9 stably accumulates in serum.** **Mice were treated with normal IgG (A-C) or 7H9 (D-F) at 1 mg/kg, i.p., QOD for 3 weeks, and serum was collected two days after the last dose. HEK293 cells were transfected with S1P_3_-GFP and incubated with diluted serum (1∶100). Detection with a Cy3-anti-mouse IgG reveals that S1P_3_-reactive antibodies are present only in 7H9-treated serum at a concentration of 10–100 µg/ml.
(TIF)

Figure S2 **Treatment with 7H9 may improve tamoxifen efficacy.** MCF7 xenograft tumors were established in a small cohort of nude mice as described in Materials and Methods. (A) Mean tumor volume was reduced by 7H9 (1 mg/kg, i.p., QOD) or tamoxifen (5 mg, 60-day release, s.q.) monotherapies, and by combined treatment. (B) Tumor cell proliferation was evaluated by immunolabeling with mitotic marker, phospho-histone H3 (EMD Millipore, Billerica, MA), and counting positive nuclei under 20X magnification. (C) Area of necrotic lesions was calculated as described in Materials and Methods. N = 3 mice/group, *p<0.05.
(TIF)
