# Supplementary figures and images for: In Vitro and In Vivo Antagonism of a G Protein-Coupled Receptor (S1P3) with a Novel Blocking Monoclonal Antibody
Source: PLoS One. 2012 Apr 5;7(4):e35129. doi: 10.1371/journal.pone.0035129 (PMC3320623; doi:10.1371/journal.pone.0035129)

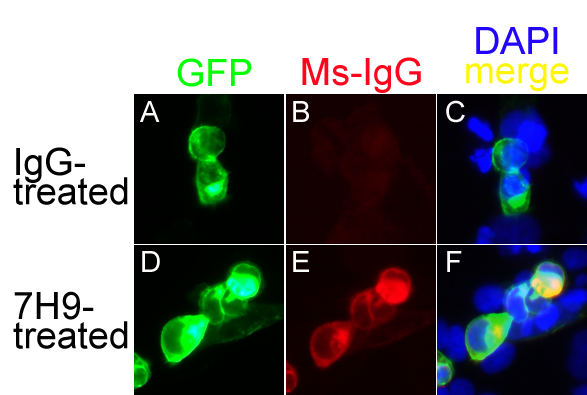

Supplement: Figure S1 — Immunocytochemical analysis of serum from treated mice demonstrates that 7H9 stably accumulates in serum. Mice were treated with normal IgG (A-C) or 7H9 (D-F) at 1 mg/kg, i.p., QOD for 3 weeks, and serum was collected two days after the last dose. HEK293 cells were transfected with S1P3-GFP and incubated with diluted serum (1∶100). Detection with a Cy3-anti-mouse IgG reveals that S1P3-reactive antibodies are present only in 7H9-treated serum at a concentration of 10–100 µg/ml. (TIF) [file pone.0035129.s001.tif]

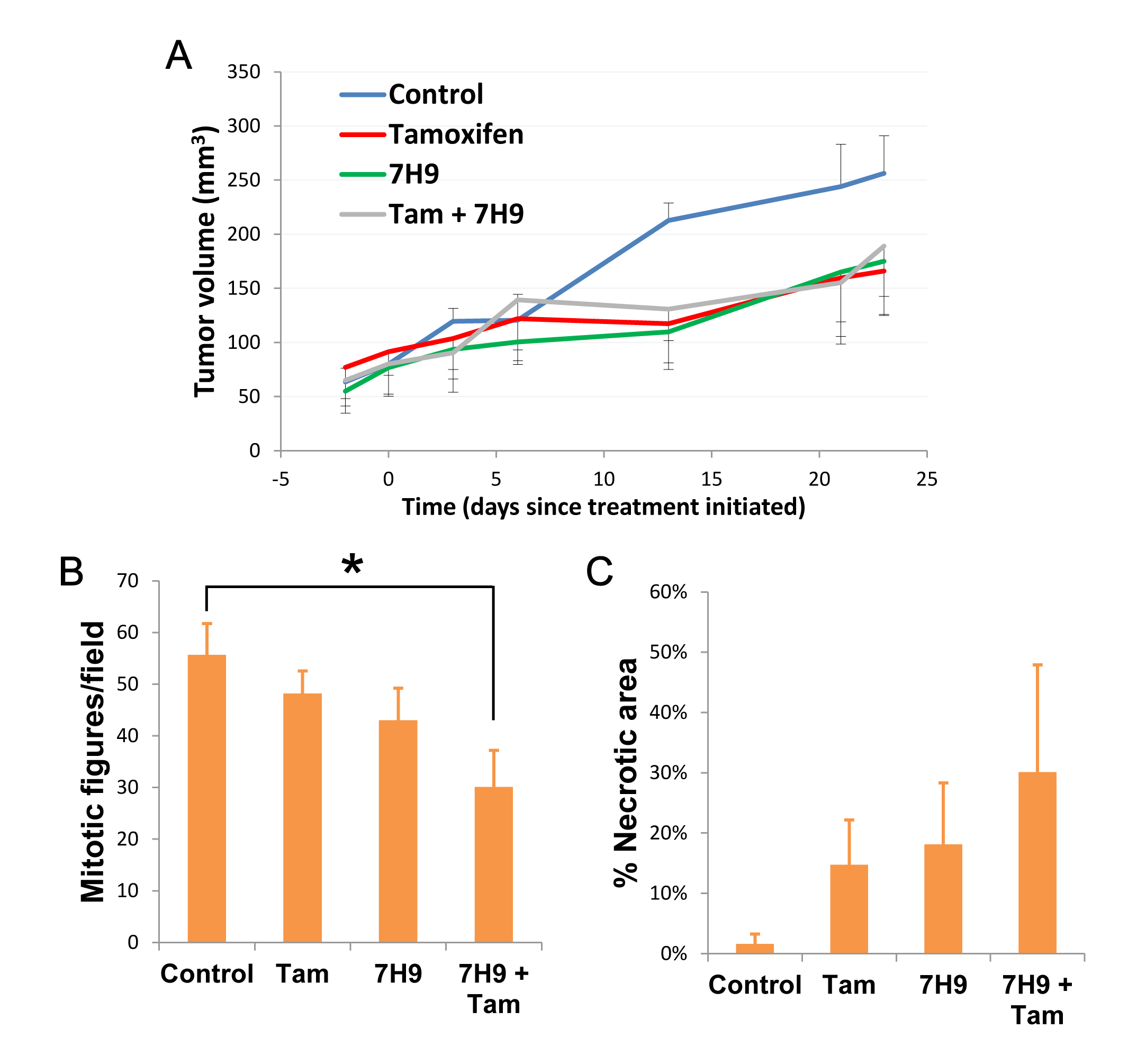

Supplement: Figure S2 — Treatment with 7H9 may improve tamoxifen efficacy. MCF7 xenograft tumors were established in a small cohort of nude mice as described in Materials and Methods. (A) Mean tumor volume was reduced by 7H9 (1 mg/kg, i.p., QOD) or tamoxifen (5 mg, 60-day release, s.q.) monotherapies, and by combined treatment. (B) Tumor cell proliferation was evaluated by immunolabeling with mitotic marker, phospho-histone H3 (EMD Millipore, Billerica, MA), and counting positive nuclei under 20X magnification. (C) Area of necrotic lesions was calculated as described in Materials and Methods. N = 3 mice/group, *p<0.05. (TIF) [file pone.0035129.s002.tif]
